# Ultra-early versus early salvage androgen deprivation therapy for post-prostatectomy biochemical recurrence in pT2-4N0M0 prostate cancer

**DOI:** 10.1186/1471-2490-14-81

**Published:** 2014-10-16

**Authors:** Satoru Taguchi, Hiroshi Fukuhara, Takeshi Azuma, Motofumi Suzuki, Tetsuya Fujimura, Tohru Nakagawa, Akira Ishikawa, Haruki Kume, Yasuhiko Igawa, Yukio Homma

**Affiliations:** Department of Urology, Graduate School of Medicine, The University of Tokyo, 7-3-1 Hongo, Bunkyo-ku, Tokyo, 113-8655 Japan

**Keywords:** Androgen deprivation therapy, Biochemical recurrence, Prostate cancer, Radical prostatectomy, Salvage androgen deprivation therapy

## Abstract

**Background:**

The optimal timing of salvage androgen deprivation therapy (ADT) for biochemical recurrence after radical prostatectomy is controversial. We compared the outcomes of ultra-early versus early salvage ADT.

**Methods:**

Among 855 patients undergoing radical prostatectomy at our institution between 2000 and 2012, we identified 121 with adjuvant-treatment-naïve pT2-4N0M0 prostate cancer who received salvage ADT for biochemical recurrence. These patients were divided into an ultra-early salvage ADT group (n = 51), who started salvage ADT before meeting the standardized definition of biochemical recurrence in Japan (two consecutive prostate-specific antigen [PSA] values ≥0.2 ng/ml), and an early salvage ADT group (n = 70) who started salvage ADT when they met the definition. The ultra-early ADT group consisted of those who started salvage ADT with a single PSA value ≥0.2 ng/ml (n = 30) or with two consecutive PSA values >0.1 ng/ml and rising (n = 21). The primary endpoint was biochemical recurrence after salvage ADT, defined as a single PSA value ≥0.2 ng/ml after PSA nadir following salvage ADT. Secondary endpoints were clinical metastasis and cancer-specific survival. A Cox proportional hazards model was used for multivariate analysis. The median follow-up was 65.5 months.

**Results:**

Biochemical recurrence occurred in one patient (2.0%) in the ultra-early group and in 12 (17.1%) in the early salvage ADT group. Multivariate analysis identified ultra-early salvage ADT and preoperative Gleason score ≤7 as independent negative predictors of biochemical recurrence after salvage ADT. Only one patient in the early salvage ADT group developed clinical metastasis to a left supraclavicular lymph node, and no patient died from prostate cancer during follow-up. The major limitations of this study were its retrospective design, selection bias, and the possibility that the ultra-early salvage ADT group may have included patients without biochemical recurrence.

**Conclusions:**

Ultra-early salvage ADT was an independent negative predictor of biochemical recurrence after salvage ADT in post-prostatectomy patients. Further consideration should be given to the use of salvage ADT before meeting the current definition of biochemical recurrence.

## Background

Approximately 25–35% of patients develop evidence of biochemical recurrence after radical prostatectomy for clinically localized prostate cancer [1, 2]. Although salvage androgen deprivation therapy (ADT) is a popular option for the management of biochemical recurrence, uncertainty remains regarding which patients benefit from early salvage ADT and the ideal time at which to initiate therapy [3]. A retrospective analysis of 1,740 patients who underwent radical prostatectomy between 1990 and 1999 found no difference in systemic progression or cancer-specific survival between men who started salvage ADT at a prostate-specific antigen (PSA) level of ≥0.4 ng/ml compared with those who did not receive salvage ADT [4]. According to a similar analysis of 1,352 patients who underwent radical prostatectomy between 1988 and 2002, early salvage ADT for biochemical recurrence after radical prostatectomy was an independent predictor of delayed clinical metastases in high-risk cases, but not in the overall cohort [5].

However, these studies were mainly conducted before the era of ultrasensitive PSA assays, since when the definition of biochemical recurrence has changed. For example, Mir et al. recently advocated definitions of biochemical recurrence as any PSA ≥0.05 ng/ml in patients with nomogram-predicted 5-year progression-free probabilities of <50% [6].

We therefore compared the outcomes of early and very early administration of salvage ADT in patients treated with radical prostatectomy for localized prostate cancer.

## Methods

We retrospectively reviewed 855 patients who underwent radical prostatectomy at our institution between 2000 and 2012 and identified 121 patients with adjuvant-treatment-naïve localized (pT2-4N0M0) prostate cancer who subsequently received continuous salvage ADT because of biochemical recurrence. Patients who underwent radiotherapy prior to or concomitant with hormonal therapy and patients who received any neo-adjuvant therapy were excluded from the study. The included patients were further divided into two groups: an ultra-early salvage ADT group (n = 51), in which patients started salvage ADT before meeting the standardized definition of post-prostatectomy biochemical recurrence in Japan (two consecutive PSA values ≥0.2 ng/ml [7]); and an early salvage ADT group (n = 70), in which patients started salvage ADT when they met the definition. The ultra-early salvage ADT group consisted of those who started salvage ADT with a single PSA value ≥0.2 ng/ml (n = 30) or with two consecutive PSA values >0.1 ng/ml and rising (n = 21). Patient allocation was not randomized. Doctors generally recommended early salvage ADT according to the General Rule for Clinical and Pathological Studies on Prostate Cancer of the Japanese Urological Association [8], but some patients were anxious and chose to have ultra-early salvage ADT. The clinicopathologic backgrounds of the two groups were similar, and the median durations of salvage ADT were 41 months in the ultra-early and 46 months in the early salvage ADT groups, respectively (Table 1). Patients who discontinued salvage ADT were treated as censored at the point of discontinuation. The median follow-up for all patients was 65.5 months (interquartile range [IQR]: 46–90.5 months) after radical prostatectomy, and an average of 33 PSA values were available per patient (i.e. total of 3,957 PSA values). We began using ultrasensitive PSA assays in September 2003.

**Table 1:** **Clinicopathologic characteristics of patients treated with ultra**-**early or early salvage ADT**

Variable	Ultra-early salvage ADT(n = 51)	Early salvage ADT(n = 70)	p-value
Median age at prostatectomy, yr (IQR)	65 (61 - 69)	67 (60.75 - 72.25)	0.2992
Age at prostatectomy, no. (%):			0.0804
<70 yr	39 (76.5)	43 (61.4)	
≥70 yr	12 (23.5)	27 (38.6)	
Median preoperative PSA, ng/ml (IQR)	10.65 (7.3 - 15.56)	9.55 (6.3 - 12.07)	0.1903
Preoperative PSA, no (%):			0.6041
<20 ng/ml	46 (90.2)	61 (87.1)	
≥20 ng/ml	5 (9.8)	9 (12.9)	
Pathologic Gleason score, no. (%):			0.3256
≤6	12 (23.5)	23 (32.9)	
7	28 (54.9)	38 (54.3)	
8-10	11 (21.6)	9 (12.9)	
Pathologic tumor stage, no. (%):			0.8619
T2	32 (62.7)	45 (64.3)	
T3/4	19 (37.3)	25 (35.7)	
Extraprostatic extension, no. (%)	17 (33.3)	25 (35.7)	0.7859
Lymphovascular invasion, no. (%)	13 (25.5)	16 (22.9)	0.7376
Positive resection margin, no. (%)	37 (72.5)	43 (61.4)	0.2019
Seminal vesicle invasion, no. (%)	2 (3.9)	2 (2.9)	0.7464
Perineural invasion, no. (%)	35 (68.6)	43 (61.4)	0.4139
Median duration time of salvage ADT, months (IQR)	41 (22 - 66)	46 (27 - 73.25)	0.1326

ADT, androgen deprivation therapy; PSA, prostate-specific antigen; IQR, interquartile range.

The primary endpoint was biochemical recurrence after salvage ADT, defined as a confirmed single PSA value ≥0.2 ng/ml after PSA nadir following salvage ADT. Secondary endpoints were clinical metastasis and cancer-specific survival. Univariate analysis was conducted using the log-rank test and multivariate analysis was performed using the Cox proportional hazards model. All statistical analyses were carried out using JMP version 9.0.2 (SAS Institute, Cary, NC, USA). A value of p < 0.05 was considered significant.

This study was approved by the Ethics Committee, Graduate School of Medicine and Faculty of Medicine, The University of Tokyo. Written informed consent for participation in this study was obtained from all participants.

As of 2014, the standardized definition of biochemical recurrence after radical prostatectomy in Japan was two consecutive PSA values ≥0.2 ng/ml at intervals of 2–4 weeks, as stated above [7], which is one of six standard definitions that have been used in published studies or are in current clinical use [6].

## Results

**Figure 1:**
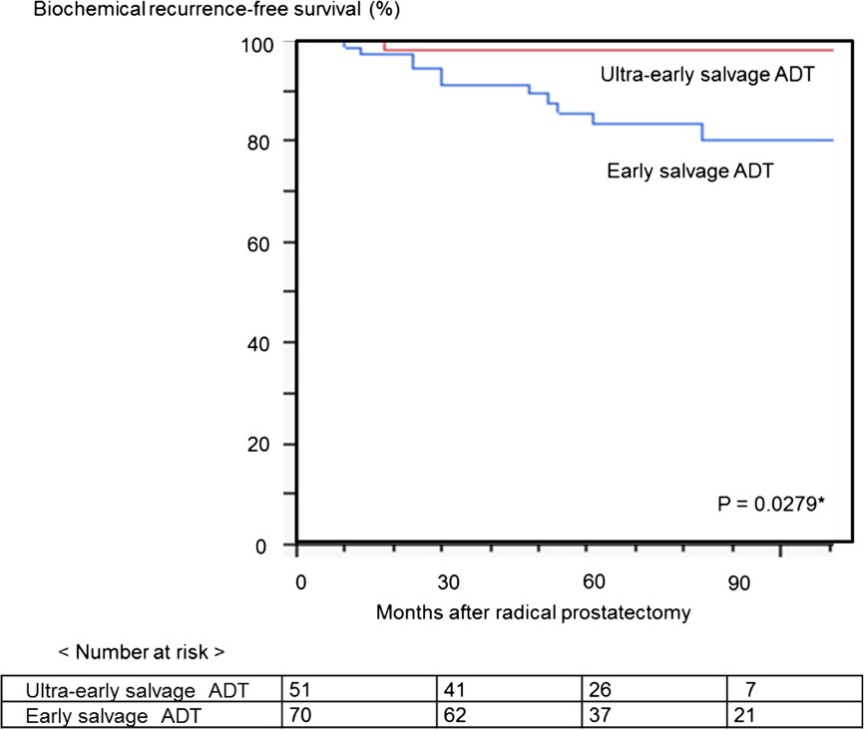
**Kaplan-Meier curve depicting biochemical recurrence-free survival in patients treated with ultra-early versus early salvage ADT.**

One of 51 (2.0%) patients in the ultra-early salvage ADT group developed biochemical recurrence, compared with 12 of 70 (17.1%) patients in the early salvage ADT group (Figure 1). Univariate analysis demonstrated that timing of treatment (ultra-early salvage ADT versus early salvage ADT, p = 0.0279), preoperative PSA value (<20 ng/ml versus ≥20 ng/ml, p = 0.0493), and pathologic Gleason score (≤7 versus ≥8, p = 0.0043) were associated with the risk of biochemical recurrence after salvage ADT (Table 2).

**Table 2:** **Univariate analysis of the impact of various clinicopathologic factors on the risk of biochemical recurrence after salvage ADT**

	No. of patients	p-value
Treatment group		0.0279*
Ultra-early salvage ADT	51	
Early salvage ADT	70	
Age, years		0.0798
<70	82	
≥70	39	
Preoperative PSA, ng/ml		0.0493*
<20	107	
≥20	14	
Pathologic Gleason score		0.0043*
≤7	101	
≥8	20	
Pathologic tumor stage		0.5690
≤2	77	
≥3	44	
Extraprostatic extension		0.7106
0	79	
1	42	
Lymphovascular invasion		0.4606
0	92	
1	29	
Positive resection margin		0.4467
0	41	
1	80	
Seminal vesicle invasion		0.3978
0	117	
1	4	
Perineural invasion		0.5035
0	43	
1	78	

ADT, androgen deprivation therapy; *statistically significant; PSA, prostate-specific antigen.

Multivariate analysis identified ultra-early salvage ADT (p = 0.0118) and preoperative Gleason score ≤7 (p = 0.0182) as independent negative predictors of biochemical recurrence after salvage ADT (Table 3).

**Table 3:** **Multivariate Cox proportional hazards regression analysis evaluating the impact of various clinicopathologic factors on the risk of biochemical recurrence after salvage ADT**

	HR(95% CI)	p-value
Treatment group		
Ultra-early salvage ADT	Reference	
Early salvage ADT	7.691 (1.466-141.6)	0.0118*
Preoperative PSA, ng/ml		
<20	Reference	
≥20	2.065 (0.529-6.761)	0.2744
Pathologic Gleason score		
≤7	Reference	
≥8	4.739 (1.330-15.73)	0.0182*

ADT, androgen deprivation therapy; *statistically significant; PSA, prostate-specific antigen.

Regarding secondary endpoints, only one patient from the early salvage ADT group developed clinical metastasis to a left supraclavicular lymph node (p = 0.4233, log-rank test), and no patient died from prostate cancer during the follow-up period. We were therefore unable to detect any differences in secondary endpoints between the two groups. This might be because the follow-up period was not long enough.

## Discussion

ADT is a well-established treatment modality for patients with advanced prostate cancer. However, despite its proven efficacy in improving the quality of life in patients with metastatic disease, there is currently no consensus regarding the optimal timing of ADT after definitive local therapy [9]. Although a small randomized trial has supported the use of adjuvant ADT after radical prostatectomy in the setting of lymph node metastases [10, 11], no randomized study has yet evaluated the utility of ADT for failure after radical prostatectomy, especially in patients without lymph node metastases [9].

Previous retrospective studies found no effect of salvage ADT on systemic progression-free survival or cancer-specific survival. Siddiqui et al. reviewed 1,740 patients who underwent radical prostatectomy between 1990 and 1999 and compared various PSA thresholds in relation to the timing of salvage ADT administration. They found no advantage in terms of systemic progression-free survival or cancer-specific survival in men who started salvage ADT at a PSA of 0.4, 1.0, or 2.0 ng/ml compared with those who did not receive salvage ADT [4]. A similar analysis by Moul et al. of 1,352 patients who underwent radical prostatectomy between 1988 and 2002 found that early salvage ADT for biochemical recurrence after radical prostatectomy was an independent predictor of delayed clinical metastases in high-risk patients (Gleason score ≥8 or PSA-doubling time ≤12 months), but had no impact on clinical metastases in the overall cohort [5].

However, these studies were mainly conducted before the era of ultrasensitive PSA assays. Ultrasensitive PSA assays allow a more precise measurement of ultrasensitive PSA values, possibly resulting in an optimal definition of biochemical recurrence after radical prostatectomy. For example, Mir et al. recently advocated the use of biochemical recurrence defined as any PSA ≥0.05 ng/ml in patients with nomogram-predicted 5-year progression-free probabilities of <50%, who might thereby benefit from early salvage radiotherapy [6]. While radiotherapy has been established as the most common salvage treatment for recurrence after radical prostatectomy in Europe and the United States, ADT still plays an important role as salvage treatment in Asia. The Asia Consensus Statement 2013 in the NCCN Clinical Practice Guidelines in Prostate Cancer states that androgen deprivation is a candidate treatment option for post-radical prostatectomy recurrence in Asian patients negative for distant metastasis [12]. Indeed, radical prostatectomy and immediate adjuvant androgen deprivation therapy achieved excellent results, including a 10-year cancer-specific survival rate of 96.3% and 10-year estimated overall survival rate of 85.7% in Japanese patients with pT3N0 prostate cancer after a median follow-up period of 8.2 years [13]. Nevertheless, studies concerning the optimal timing of salvage ADT in the era of ultrasensitive PSA assays are lacking.

In the present study, patients who started salvage ADT before meeting a currently accepted definition of biochemical recurrence were less likely to develop subsequent biochemical recurrence after salvage ADT than those who started salvage ADT when they met the definition. By its very nature, ADT is less likely to contribute to local control than radiotherapy. However, ADT may improve local control when the residual tumor burden is very small, and adjuvant ADT after radical prostatectomy improved the clinical outcome in patients with adverse pathologic findings [10, 11]. In a randomized prospective controlled trial, Messing et al. demonstrated that adjuvant ADT benefited patients with nodal metastases who had undergone prostatectomy and lymphadenectomy, compared with those who receive deferred treatment [10, 11]. Referring to the results of European Organization for Research and Treatment of Cancer (EORTC) trial 30846, which found no benefit of immediate ADT compared with deferred therapy in men without definitive local treatment of the primary tumor [14], Messing et al. concluded that ADT was probably most effective against very small tumors [10, 11, 15]. The estimated tumor burden in EORTC 30846 was ≥12.5 cm^3^ of cancer tissue, which was the mean local-tumor volume in Messing et al.’s patients [11]. Several experimental systems have provided collateral evidence to support the benefit of early ADT in inhibiting tumorigenesis [16–18].

A similar concept was recently demonstrated in the setting of salvage radiotherapy. Briganti et al. reported that timely administration of early salvage radiotherapy (given at PSA ≤0.5 ng/ml) was comparable to adjuvant radiotherapy for improving biochemical recurrence-free survival in pT3N0 prostate cancer, and suggested that initial observation followed by early salvage radiotherapy delivered at low PSA levels might be a viable option for the majority of surgically-managed patients with pT3N0 prostate cancer [19]. The present study may imply that we should consider using a PSA threshold below the currently accepted definition of biochemical recurrence in order to maximize the benefit from salvage ADT. Together with an excellent result of adjuvant ADT post-prostatectomy against pT3N0 prostate cancer [13], ADT might be more efficacious than other treatments in small tumors. However, ADT is associated with some real risks related to metabolic syndrome.

Our study had several limitations. The ultra-early salvage ADT group may have included patients without evidence of biochemical recurrence, which may have resulted in overestimation of the outcomes of ultra-early salvage ADT. Other limitations were its retrospective design, selection bias, lead-time bias, small sample size, and the inclusion of only Japanese subjects. Randomized prospective studies with longer follow-up periods are thus needed to confirm the benefits of ultra-early salvage ADT.

## Conclusions

Ultra-early salvage ADT is an independent negative predictor of biochemical recurrence after salvage ADT in adjuvant-treatment-naïve pT2-4 pN0 M0 radical prostatectomy patients. The currently accepted definition of biochemical recurrence should be challenged in relation to the optimal timing of initiating salvage ADT.

## Abbreviations

ADT: Androgen deprivation therapy
PSA: Prostate-specific antigen
IQR: Interquartile range
EORTC: European Organization for Research and Treatment of Cancer.
